# Vulnerabilities of people with different types of disabilities in disasters: a rapid evidence review and qualitative research

**DOI:** 10.1111/disa.12686

**Published:** 2025-05-12

**Authors:** Kien Nguyen‐Trung, Trinh Thi Thu Thuy, Nguyen Phuong Anh, Ngo Cong‐Lem, Do Thi Huyen, Le Thi Diu, Nguyen Hong Giang, Michael Simon

**Affiliations:** ^1^ Water Sensitive Cities Australia, Monash Sustainable Development Institute Monash University Australia; ^2^ Hanoi Association of People with Disabilities Vietnam; ^3^ Faculty of Foreign Languages Dalat University Vietnam

**Keywords:** climate change, disaster, extreme weather events, people with disabilities, rapid evidence review, thematic analysis, vulnerability

## Abstract

Despite the growth of disaster scholarship, the topic of how and why climate‐related disasters and extreme weather events vary among people with different types of disabilities remains unexplored. To help fill the gap, this study draws on a larger research project that was co‐designed by Water Sensitive Cities Australia at Monash University and the Hanoi Association of People with Disabilities, Vietnam. It utilised the dataset of a rapid evidence review of 33 studies, key informant interviews with 26 local stakeholders, and 52 interviews with people with various disabilities in Hanoi and Nghe An province, Vietnam. Using thematic analysis, we identified eight themes pertaining to socially‐constructed difficulties facing people with disabilities: barriers to accessing disaster risk information and warnings; difficulties in understanding emergencies; challenges in communicating needs; evacuation and mobility hurdles; decreased sense of belonging and isolation; increased risk of getting sick; increased risk of developing mental health and behavioural disorders; and disrupted livelihood and loss of income.

## 1 INTRODUCTION

Vietnam is among the world's most disaster‐prone countries, facing severe risks from climate change and natural hazards. Ranked 91st out of 191 countries in terms of disaster risk, the nation is particularly vulnerable to flooding, tropical cyclones, and droughts. Coastal and river delta regions are at heightened risk due to sea‐level rise and storm surges, with projections suggesting that between 6 and 12 million people could be affected by coastal flooding by the end of the twenty‐first century (World Bank and Asian Development Bank, 2020, p. 2). Rising temperatures will further compound these challenges, leading to chronic heat stress, particularly among poorer communities—temperatures are expected to increase by 1.0–3.4°C (degrees Celsius) by 2080–99 in comparison to the 1986–2005 baseline (World Bank and Asian Development Bank, 2020, p. 2).

People with disabilities bear a disproportionate share of the negative impacts of climate change and disaster risks (Alexander, Gaillard, and Wisner, 2012). They comprise approximately seven per cent of Vietnam's population (about 93 million according to the latest national survey, in 2016), with a significant majority of its members being women and children (GSO, 2016). Despite their sizable presence, people with disabilities are often marginalised in disaster risk reduction (DRR) and climate change adaptation policies (Ton et al., 2020) and have been largely overlooked in disaster risk management and climate change adaptation strategies (UN Women, 2022).

The specific challenges that people with disabilities face before, during, and after disasters and extreme weather events (EWEs) remain under‐researched, especially in the context of Vietnam. During disasters/EWEs, they confront substantial difficulties, particularly barriers to accessing emergency information and navigating evacuation routes. For example, individuals with hearing impairments may miss crucial emergency warnings if they are not delivered in accessible formats, such as the use of sign language or captioned broadcasts (Habibisaravi et al., 2022). Similarly, visually‐impaired individuals may struggle to avoid hazards, especially if they lose access to assistive devices including canes or guide dogs (Stough, 2009). During an evacuation, people with disabilities often encounter inadequate amenities at a shelter, such as a lack of ramps and accessible bathrooms (Gartrell et al., 2020). Moreover, they are more likely to experience social discrimination and, in some cases, sexual violence in these settings (Battle, 2015; Mendis et al., 2023).

Beyond disasters/EWEs, people with disabilities are often excluded from disaster preparedness training and emergency planning, which leaves them less prepared for crises. Their generally lower socioeconomic status further hinders access to education and emergency resources (Craig et al., 2019; Calgaro et al., 2021; Lindsay and Hsu, 2024). Additionally, many people with disabilities reside in substandard housing, which is vulnerable to collapse during EWEs or earthquakes (Stough and Kelman, 2018; King et al., 2019). These issues increase their risk of injury or death during disasters/EWEs (Alexander, Gaillard, and Wisner, 2012; Ronoh, Gaillard, and Marlowe, 2015). Recovery is further complicated by the disproportionate allocation of resources, as people with disabilities receive less despite bearing a greater financial burden relative to their income (Stough and Kelman, 2018).

Existing studies have largely focused on mobility impairments, overlooking other forms of disability such as hearing, visual, or cognitive impairments (Stough, 2009; Gaskin et al., 2017). For example, while it is well‐documented that individuals with mobility impairments face problems in evacuating quickly or avoiding hazards like falling debris (Aryankhesal, Pakjouei, and Kamali, 2018), research on the difficulties encountered by those with cognitive impairments in terms of understanding disaster situations or making timely decisions is sparse. Likewise, the compounding conditions of people with multiple disabilities (such as those with both intellectual and mobility disabilities) remain largely unexplored. Moreover, there is a lack of scholarship that compares and contrasts the vulnerabilities of people with various types of disabilities in the sphere of disasters. These gaps highlight the need for more comprehensive research that considers the full spectrum of disabilities and associated vulnerabilities during disasters.

This paper seeks to fill these lacunae by investigating the following research question: how and why do the impacts of climate‐related disasters and EWEs vary among individuals with different types of disabilities in the context of Vietnam? To do so, we base our paper on a dataset from a larger research project that was co‐designed by Water Sensitive Cities Australia at Monash University and the Hanoi Association of People with Disabilities, Vietnam (DP Hanoi)—a people with disabilities‐led organisation in Vietnam. Our methodology combines a rapid evidence review with key informant interviews (KIIs) and interviews with people with disabilities in Hanoi, the capital of Vietnam, and Nghe An province.

The paper starts with a description of our research methodology, before presenting themes pertaining to the challenges faced by people with different types of disabilities, and then discussing the contribution of the work to the current literature.

## 2 CO‐DESIGN AND MULTIPLE METHODS APPROACH

### 2.1 Overview of the research approach

Advocacy by DP Hanoi for the incorporation of disability inclusion principles in water laws reform in Vietnam led to this study. As the topic of climate‐related disasters and disability and social inclusion was relatively underdeveloped, DP Hanoi was in urgent need of related evidence to inform its policy‐oriented advocacy. In this context, the Australian Water Partnership (AWP) funded a timely project that enabled collaboration between Water Sensitive Cities Australia and DP Hanoi to explore the vulnerabilities of and challenges to people with disabilities in Vietnam with respect to climate change, water insecurity, and disasters/EWEs (Nguyen‐Trung et al., 2024).

In alignment with the ‘nothing about us without us’ motto, this project was designed as equitable and experientially‐informed research, aiming to address equality, diversity, and disability inclusion in climate change, water security, and disaster risk management (Smith et al., 2023, p. 164). This approach prioritises the participation of partners with disabilities (that is, DP Hanoi) whose lived experience is of prime importance in understanding and interpreting the challenges posed to people with disabilities in Vietnam by climate change, water insecurity, and disaster risks.

DP Hanoi is an organisation of and for people with disabilities in Hanoi, comprising practitioners and staff with disabilities. They have lived experience of disability inclusion in and vulnerability to climate change, water insecurity, and disaster risks and have established partnerships with many organisations for people with disabilities (OPDs) in the country. This allowed the research team to refine better the research question, design the research process, collect, analyse, and interpret data, and generate recommendations. The equal participation of DP Hanoi's staff and practitioners helped academic partners such as Water Sensitive Cities Australia and BehaviourWorks Australia at Monash University to navigate Vietnam's contextual complexity and to address the unequal power relations that people with disabilities often confront in social research in Vietnam. Our action‐learning approach meant that all partners exchanged knowledge and experience throughout the project cycle, from workshop and training sessions to co‐data gathering and co‐analysis.

**FIGURE 1:**
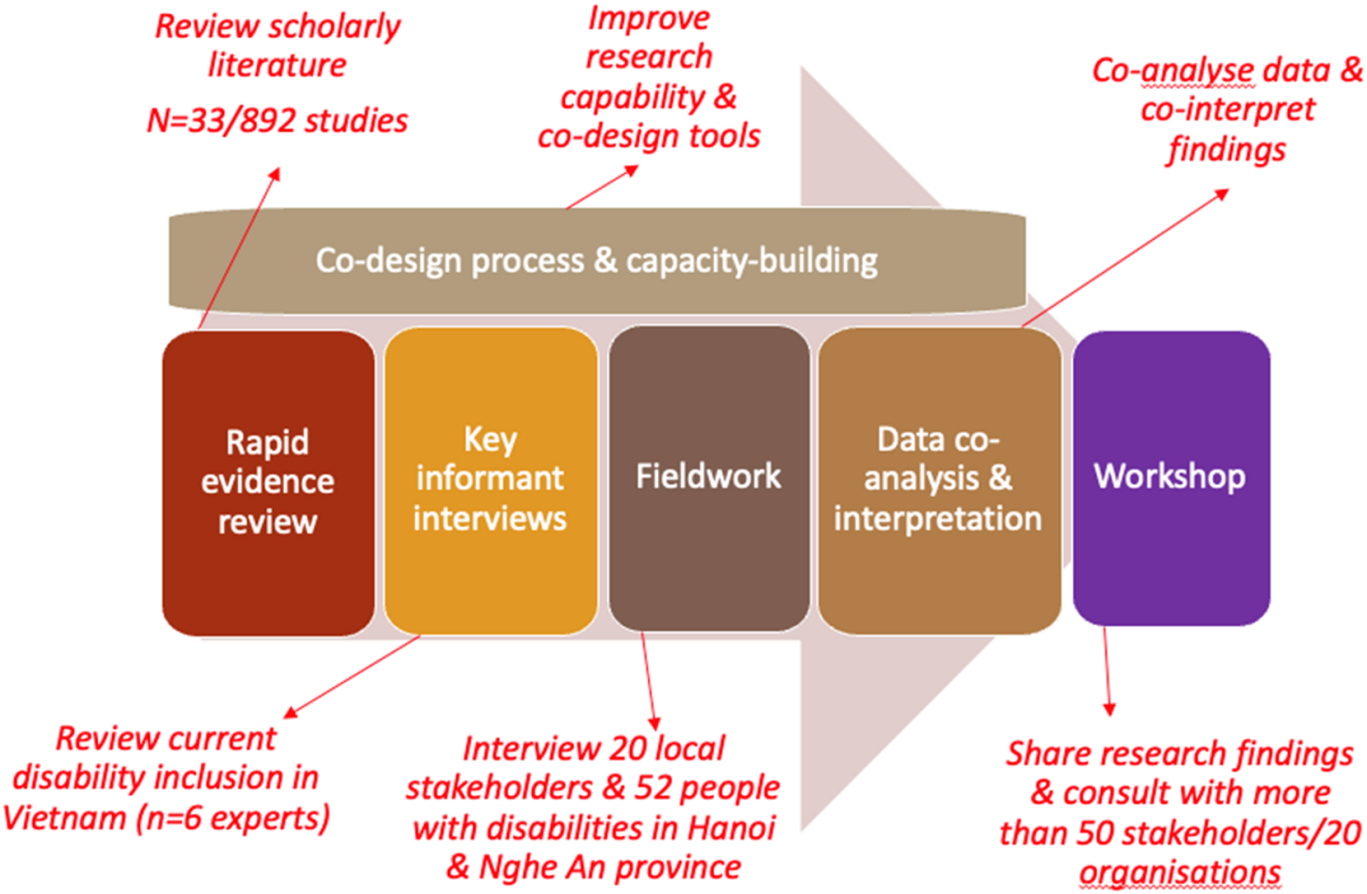
The research process. **Source:** authors.

This is a qualitatively‐driven multiple methods research project with ethical approval of the Monash University Human Research Ethics Committee (received on 18 October 2023). Figure 1 showcases our research process, whereby we combined different methods to understand the research topics: a rapid evidence review, KIIs with local stakeholders, and semi‐structured interviews with people with disabilities, as well as a consultative workshop with different parties. Throughout this process, we employed a co‐design research and capacity‐building approach: Water Sensitive Cities Australia helped to improve the DP Hanoi team's research capacity and in return, DP Hanoi taught Water Sensitive Cities Australia about disability inclusion in Vietnam. Water Sensitive Cities Australia organised six training sessions: one was conducted online via Zoom and five took place in person in Hanoi. The training involved 14 participants from DP Hanoi and its partners, of which 10 were people with disabilities (Nguyen‐Trung et al., 2024).

The research project started with a rapid evidence review and KIIs. The former helped to procure scholarly evidence at the intersection of climate change and disasters/EWEs, water insecurity, and disability inclusion, whereas the latter, 26 in total including six with experts at the central/national level, helped to paint an overview picture of disability inclusion in Vietnam. Both of these methods played a supplementary role in establishing the key assumptions that led to the co‐design of primary data collection with people with disabilities. During fieldwork, we combined KIIs with stakeholders (such as local authorities) that sought information on disability inclusion in relation to climate change and disaster risk management and semi‐structured interviews with people with disabilities at two sites in Hanoi and Nghe An province. The objective was to compare and contrast the information with the assumptions secured from the rapid evidence review. Lastly, a workshop in Hanoi in January 2024 allowed the research team to share its findings with national and international stakeholders and organisations.

### 2.2 A rapid evidence review

A rapid evidence review, or rapid evidence assessment, applies the principles of systematic review—the gold standard in evidence synthesis—to a faster review process that meets the urgent demands of decision‐making in a shorter period of time (Abboah‐Offei et al., 2021). This approach allows researchers to limit their search and review (such as in terms of number of databases and reviewers) to address time and resource constraints (Speckemeier et al., 2022). Following the recommendations of Bragge et al. (2023), we designed our rapid evidence review as a supplemental tool for our qualitative fieldwork (that is, the semi‐structured interviews with people with disabilities). Consequently, it had to be conducted under a tight timeline from late September to the end of October 2023 to garner key insights for the fieldwork.

The review was developed in collaboration with the BehaviourWorks Australia team members, who have expertise in evidence‐reviewing services. In consultation with a librarian specialist, we developed a review protocol (see Appendixes 1, 2, and 3 in the supplementary materials)^1^ to address the requirements and limitations in terms of time and resources. Our rapid evidence review aimed to answer the previously stated research question: how and why do the impacts of climate‐related disasters and EWEs vary among individuals with different types of disabilities in the context of Vietnam? It focused only on two databases: Scopus and Google Scholar. In the former, we narrowed our search to the roughly six‐year period between 2017 and 16 October 2023, whereas in the latter, we screened only the first 105 citations. Screening and data extraction were conducted by one review specialist, while quality appraisal was omitted owing to resource limitations.

These constraints introduced potential biases into the review. To respond to this, we cross‐checked the findings with data gathered during the fieldwork. The combination of the rapid evidence review and the intensive fieldwork with multiple stakeholders and people with disabilities allowed us to refine our insights. Although similar to an evidence and practical review (Bragge et al., 2023), our approach included more extensive fieldwork to strengthen the reliability of the study's conclusions.

### 2.3 Key informant interviews

The KIIs were designed as semi‐structured interviews. A guide was co‐developed by Vietnamese interviewers from DP Hanoi and Water Sensitive Cities Australia to guide the interviews with stakeholders. Including the experts, 26 key informants made up the total sample (including 10 people with disabilities and 11 females). Key informants are classified as experts at the central/national level and local stakeholders. Experts were purposefully chosen based on the following criteria: (i) they were interested in the topics under review; and (ii) they were principal players in the field of disability inclusion who could actively contribute to the consultative workshop in January 2024, which aimed to influence Vietnam's policies on disability inclusion. Six in total were selected at this level, comprising a central government official, researchers, and leaders of OPDs and international organisations in Vietnam.

Key informants at the local level were purposefully selected based on the following criteria: (i) they were primary stakeholders who were crucial to local disability inclusion, climate change adaptation, disaster risk management, and water resources management; (ii) they could diversify our understanding of the topics; (iii) and they could represent local people with disabilities. Eventually, 20 key informants were identified in Hanoi and Nghe An province: 10 were leaders of OPDs, five were leaders of a Commune or a Ward People's Committee, one was the leader of a people with disabilities‐led Cooperative, and four were leaders of sociopolitical organisations.

### 2.4 Interviews with people with disabilities

Interviews with people with disabilities was the main source of empirical data. We conducted 52 semi‐structured interviews across Cau Giay and My Duc districts in Hanoi and Vinh city and Thanh Chuong district in Nghe An province (see Table 1). The aim was to collect information from people with disabilities in both urban and rural settings, as well as in a range of social contexts and areas of climate change vulnerability. The fieldwork team members were all Vietnamese (including one researcher from Water Sensitive Cities Australia), and most (four out of five) have a certain type of disability. Interviews were carried out in Vietnamese, and all of the data were gathered and analysed in the same language, leaving a legacy resource for DP Hanoi and its OPD members. This method focused on understanding people with disabilities' experiences and challenges relating to natural hazards and climate change. The data collected included quantitative indicators (gender, age, self‐claimed types of disabilities, and level of difficulty in carrying out different daily activities) and qualitative information, including perceptions of climate change and experiences of EWEs (see Appendix 5 in the supplementary materials).

**TABLE 1:** Profiles of people with disabilities.

Type of disability	Location				Total
	Hanoi		Nghe An province		
	Cau Giay district	My Duc district	Vinh city	Thanh Chuong district	
Vision	3	2	2	2	9
Hearing	3	–	2	2	7
Mobility	2	6	5	2	15
Intellectual	0	1	2	5	8
Multiple	6	2	2	3	13
Age group					
18–40	8	4	6	7	25
41–59	4	6	5	4	19
60+	2	1	2	3	8
Total	14	11	13	14	52
Sex					
Male	9	7	6	8	30
Female	5	4	7	6	22
Total	14	11	13	14	52

**Source:** Nguyen‐Trung et al. (2024).

**FIGURE 2:**
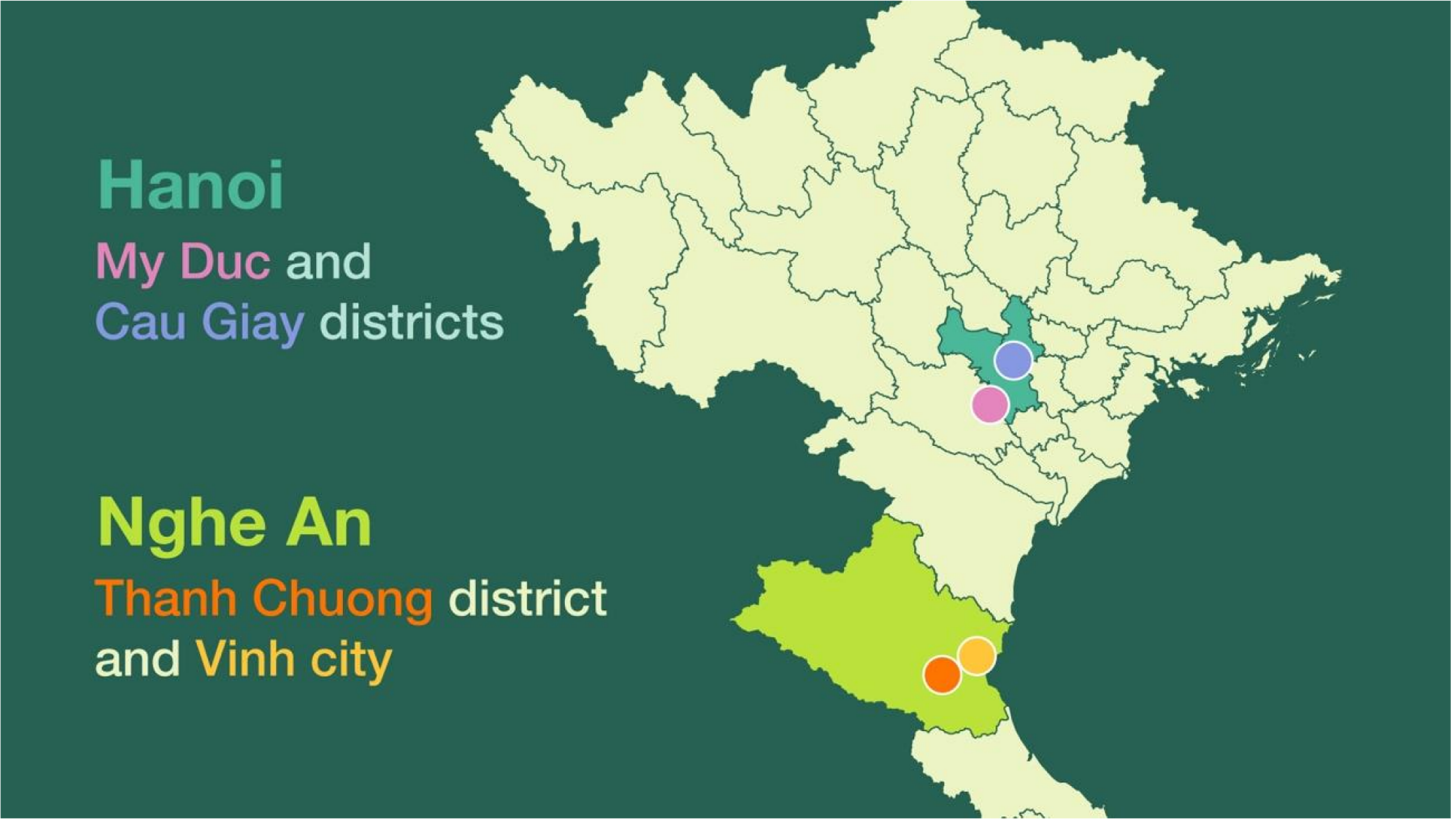
The research sites. **Source:** Nguyen‐Trung et al. (2024).

The fieldwork was conducted in the same four research sites. Cau Giay and Vinh city are urban areas, whereas My Duc and Thanh Chuong are rural areas (see Figure 2). The selection of these sites was based on two key reasons presented by DP Hanoi and its partners. First, they are DP Hanoi's main working areas; hence, it had strong partnerships with local stakeholders (including local authorities and OPDs) that could potentially support the recruitment of people with disabilities there. As the time frame for data collection was limited to 18 October (after the ethics application was approved by Monash University) to November 2023 (before upcoming holidays in Vietnam and Australia), having strong local partnerships was crucial. Second, Hanoi and Nghe An province are both vulnerable to natural hazards; however, by the time of the project, there was a lack of data informing policies regarding the vulnerability of people with disabilities to climate change and disasters/EWEs in these research sites. The gathering of empirical data made a significant contribution to DP Hanoi's current policy advocacy.

Hanoi is located in the northern region of Vietnam. Comprising one city, 12 urban districts, and 17 suburban districts, its estimated population in 2019 was around 8.4 million people, with the urban population accounting for approximately 49.1 per cent of the total population. The average income per capita is VND 6,423,000 per person per month (GSO, 2023). Cau Giay, an urban district consisting of eight wards, spans an area of 12.38 square kilometres (km^2^) and has a population of 294,500 (as of 2022), whereas My Duc, a suburban district comprising 21 wards/communes, is located in the southern part of the capital, encompassing an area of 226.31 km^2^ and with a population of 210,200 (Hanoi Statistics Office, 2023).

Nghe An province, meanwhile, is in the north‐central region of Vietnam. It is bordered by Thanh Hoa province to the north and Ha Tinh province to the south and it is the country's largest province, covering an area of 16,486.49 km^2^. With 21 district‐level units, including one city, three towns, and 17 districts, Nghe An's population is 3.4 million, comprising approximately 490,000 (14.3 per cent) urban residents and more than 2.9 million (85.7 per cent) rural residents. Vinh, the city in Nghe An, is located in the coastal zone, spanning an area of 105 km^2^ and with a population of 349,206 (as of 2022), whereas Thanh Chuong, an upland and mountainous district in the southern part of the province, covers an area of 1,126.93 km^2^ and has a total population of 245,551 (as of 2022) (Nghe An Statistics Office, 2022).

Hanoi has 116,133 people with disabilities (accounting for 1.39 per cent of the population), of which females constitute 47.4 per cent: 77,001 individuals with ‘severe’ disabilities, 20,227 with ‘mild’ disabilities, and 18,905 with ‘very severe’ disabilities (Hanoi Statistics Office, 2023). The Nghe An Province Association for People with Disabilities, established in 2017, currently has more than 1,200 members, and has established two district‐level associations, five branch associations in districts, six women with disabilities clubs, and five disability clubs. Thanh Chuong district has not yet established a disability association (Minh, 2023).

In this research, we purposefully employed a stratified sampling strategy (Patton, 2002) informed by a case‐oriented approach (Yin, 2014), allowing us to choose two different location sites, namely, Hanoi and Nghe An province. In each of these locations, we chose two districts representing differences between urban and rural areas in terms of how climate change impacts disability inclusion. We purposefully selected people with disabilities based on their vulnerability to climate change, water access, and disasters/EWEs (see Appendix 4 in the supplementary materials for more information). Table 1 presents the profiles of the participants.

### 2.5 Data management and analysis

Data management and analysis (carried out from November 2023–January 2024) provided the outputs for the consultative workshop on 10 January 2024. DP Hanoi researchers took the lead on transcribing interview records and arranging and storing data in applications such as Google Drive, Google Sheets, and Google Forms. All data collected will be stored for a minimum of five years on Monash University's server in compliance with state legislation and to ensure sufficient time for the research to be referenced. DP Hanoi can access this data and use it for policy advocacy.

We employed a template analysis approach (King, Brooks, and Tabari, 2018; King, Horrocks, and Brooks, 2019) to facilitate data analysis. The rapid evidence review produced eight themes concerning the challenges facing people with different types of disabilities in disasters/EWEs. We used these a priori themes principally to guide our coding of the qualitative data. The DP Hanoi research team (composed of four people with disabilities and one staff member with no disability) were involved in reading, coding, clustering, developing, and interpreting the themes. The team started preliminary coding by assigning two members to code independently the first transcripts. It compared its lists of codes to generate the initial form of a coding scheme or template. Then the team divided the remaining transcripts and used the initial template to code them. Next, it engaged in analysis by comparing and contrasting the rapid evidence review and the qualitative themes. The latter provided a detailed, contextual explanation of how and why participants with certain types of disabilities face the hurdles pinpointed in the review.

## 3 FINDINGS

In this section, we attempt to mix the results of the rapid evidence review and the qualitative fieldwork. To do so, we first present the themes concerning the challenges faced by people with disabilities in disasters/EWEs identified by the review and then use our qualitative findings to further explore the issues.

### 3.1 Challenges faced by people with different types of disabilities

Based on the rapid evidence review, we were able to document how people with different types of disabilities face unique impediments in disasters/EWEs (see Table 2). Accordingly, people with mobility disabilities often find themselves vulnerable during the evacuation process and when seeking shelter. Common reasons are that they experience difficulties in navigating obstacles and narrow spaces (Taylor et al., 2022), they are unable to use the lift to go downstairs, and they lack a disability supporter or appropriate transportation equipment (Park, Yoon, and Choi, 2019). For people with hearing disabilities, hardships are frequently derived from their dependence on assistive devices such as hearing aids (King et al., 2019), having to express their needs to rescue teams, and a lack of access to public sound‐based alerts and real‐time emergency notifications in sign language (Cooper et al., 2021). People with visual disabilities also face the challenge of gaining access to emergency messages, but this stems from their inability to see what is happening during a cyclone, for instance, or their high dependence on accompanying support (Quaill, Barker, and West, 2019). People with cognitive or intellectual disabilities often struggle to make sense of surrounding events and changes in emergency situations (King et al., 2019) and encounter increasing distress and aggression (Stough and Kelman, 2018).

**TABLE 2:** Challenges faced by people with different types of disabilities during disasters/EWEs.

Disability type	Challenges	Key citations
Mobility	Difficulty evacuating and seeking shelter, hindered by obstacles and narrow spaces, vulnerable to small particles, disruption of daily life, isolation, and dissatisfaction.	Van Willigen et al. (2002); Bourke et al. (2017); Aryankhesal, Pakjouei, and Kamali, (2018); Park, Yoon, and Choi (2019); Taylor et al. (2022).
Hearing	Difficulty accessing emergency information, difficulty communicating with rescue teams, failure to evacuate in a timely manner, and losing assistive devices.	King et al. (2019); Cooper et al. (2021); Habibisaravi et al. (2022).
Visual	Difficulty navigating, inability to access assistive devices, a lack of access to emergency messages, not knowing how to evacuate, and vulnerable to sexual violence.	Stough (2009); Park, Yoon, and Choi (2019); Quaill, Barker, and West (2019); Jodoin, Ananthamoorthy, and Lofts (2020); Ssennoga et al. (2022).
Cognitive or intellectual	Difficulty understanding surrounding events, poor judgement, becoming agitated and losing control in crises, and a lack of medical care.	Stough (2009); Alexander, Gaillard, and Wisner (2012); Ronoh, Gaillard, and Marlowe (2015); Gaskin et al. (2017); Stough and Kelman (2018).

**Source:** authors (rapid evidence review).

### 3.2 Challenges faced by people with different types of disabilities: findings from the rapid evidence review and fieldwork

The rapid evidence review's results provided us with key insights into the hurdles facing people with different types of disabilities during disasters/EWEs. These insights formed our assumptions and guided our interviews with people with disabilities and key informants at the local level. Table 3 presents our attempts to examine these principal assumptions by providing contextual information from the qualitative interviews. The first column presents eight ‘themes’ representing the types of challenges faced by people with different types of disabilities—one should note that these challenges do not apply to all types of disabilities. ‘Yes’ in the ‘review’ columns indicates that a specific type of challenge was discussed in direct relation to certain types of disability in the studies we reviewed. Blank cells in the same columns show that, although a particular challenge might be relevant to people with disabilities generally, the literature we assessed did not specify it as affecting particular types of disabilities. ‘Yes’ in the ‘fieldwork’ columns indicates that this theme was confirmed by the data. A special case arose with themes related to mental and physical health impacts: these challenges were identified primarily in relation to individuals with mobility disabilities and mental health impairments; however, we also uncovered these challenges among individuals with visual impairments.

**TABLE 3:** Comparison of the challenges faced by people with different types of disabilities in disasters/EWEs between, rapid evidence review and fieldwork data.

Themes	Mobility disabilities		Visual disabilities		Hearing disabilities		Cognitive disabilities	
	Review	Fieldwork	Review	Fieldwork	Review	Fieldwork	Review	Fieldwork
Barriers to accessing disaster risk information and warnings	–	–	Yes	Yes	Yes	Yes	–	–
Difficulties in understanding emergencies	–	–	–	–	–	–	Yes	Yes
Challenges in communicating needs	–	–	–	–	Yes	Yes	Yes	Yes
Evacuation and mobility hurdles	Yes	Yes	Yes	Yes	–	–	Yes	Yes
Decreased sense of belonging and isolation	Yes	Yes	Yes	Yes	Yes	Yes	–	–
Increased risk of getting sick	Yes	Yes	–	Yes	–	–	Yes	Yes
Increased risk of developing mental health and behavioural disorders	–	Yes	–	–	–	–	Yes	Yes
Disrupted livelihood and loss of income	Yes	Yes	–	Yes	–	Yes	–	–

**Note:** cognitive disabilities here include people with mental health impairments and intellectual disabilities.

**Source:** authors.

#### 3.2.1 Barriers to accessing disaster risk information and warnings

An early warning is crucial in disaster situations. Yet, the literature review indicates a gap in accessible media and communication products, particularly for people with visual impairments (inadequate radio broadcast quality and a lack of weather narration) and those with hearing impairments (public sound‐based alerts and the absence of real‐time emergency notifications in sign language) (Cooper et al., 2021; Gomes, Marchezini, and Sato, 2022).

Deaf people indicated in our interviews that they predominantly relied on interpersonal relationships and family members for alerts, rather than technological means and government announcements. Vietnamese television programmes such as VTV2 News have sign language interpretation running in parallel to narration of disaster warnings and weather forecasts by a reporter; however, this type of translation still needs to be more user‐friendly from a disability standpoint. A 38‐year‐old male with hearing and speech disabilities (PH22) shared his experience:

*TV programmes, especially weather forecasts and community communications for people with disabilities, need to enlarge the frame of the sign language interpreter. The programme [VTV2 News] aired at 22:00 . . . [it] should be scheduled at more appropriate, earlier times so that deaf people can access them. Not everyone lives in the city and has the conditions to watch. People in rural areas usually don't stay up late. They will watch whenever the family watches*.

The presence of sign language interpreters on channels like VTV is positive, but implementation falls short since not all deaf individuals, especially those living in rural areas such as Thanh Chuong district, Nghe An province, and My Duc district, Hanoi, are proficient in sign language. A 19‐year‐old male with hearing disabilities in Vinh city (PA19) expressed his desire to learn sign language: ‘I really wish I could go to school to learn about culture and sign language, and I hope my family members can learn it too. It would make understanding each other and daily life so much better’. Interviews with local authorities in Nghe An province confirmed the point. For instance, a social protection officer (KII18) commented: ‘In Vinh city and Nghe An, most deaf people struggle with learning sign language due to a lack of teachers/classes. There's no specific support policy for people with disabilities during disasters’.

Meanwhile, visual formats prevalent on television pose a considerable barrier to individuals with visual impairments, as they typically rely on visual cues like images and graphics, often accompanied by music rather than verbally descriptive narration. A 42‐year‐old man with a vision disability from Vinh city (PA10) expressed frustration with weather forecasts on television, highlighting that while they are detailed, specific areas are not clearly indicated, and the visual content is too small to be accessible by visually impaired individuals. Rural participants with visual impairments underlined their reliance on loudspeakers, village officials, and family members. A 52‐year‐old man with visual disabilities, from My Duc district, Hanoi (PH23), shared his reliance on his child to relay information, indicating that current forecasts' dependence on visual elements without accompanying narration renders them inaccessible by someone who cannot see. Meanwhile, some urban participants in My Duc have found alternatives through the use of smartphones and social media platforms such as Facebook and Zalo, along with apps specifically designed for news consumption by the visually impaired (PH24, My Duc, male with vision disabilities, 59 years old).

#### 3.2.2 Difficulties in understanding emergencies

For people with intellectual or cognitive disabilities, the main challenge is not just having access to disaster risk warnings, but more importantly, making sense of emergency situations so as to take timely decisions. The rapid evidence review reveals that cognitive impairments lead to difficulties in recognising emergencies (Gaskin et al., 2017) and assessing danger levels (King et al., 2019), and they may result in poor judgement and decision‐making, increasing the possibility of injury in crisis situations (Alexander, Gaillard, and Wisner, 2012; Jodoin, Ananthamoorthy, and Lofts, 2020).

During emergencies, individuals with cognitive disabilities in Hanoi and Nghe An province have a profound dependence on family members for safety and guidance owing to their inability to make their own decisions. This reliance was vividly illustrated by a mother in Thanh Chuong district, Nghe An province (PA18), who described the process of evacuating her two daughters with intellectual disabilities to safety during floods:

*When natural hazards occurred, we needed to move to a safe place, they [her two intellectually disabled daughters] relied entirely on my support since they didn't know anything. I had to take them down to the People's Committee building [temporary shelter]*.

Making timely decisions and taking early action are deemed harder for individuals with multiple disabilities. A 52‐year‐old man living in Nghe An province who was born with intellectual, hearing, and mobility disabilities is entirely reliant on his wife's support. She (PA21) noted: ‘Our family doesn't have a TV or radio.. .. The village loudspeaker does announce [the disaster risks], but sometimes I am busy working and can't hear the announcement. He always stays at home and can't hear anything’.

#### 3.2.3 Challenges in communicating needs

People with hearing and cognitive disabilities were found to be vulnerable during disasters/EWEs owing to their inability to express their needs. Calgaro et al. (2021) revealed that deaf people in Australia have experienced difficulties in communicating with emergency responders (including shelter staff), which usually requires sign language interpreters. Our fieldwork found no explicit information regarding rescue teams or responders, but some deaf people interviewed faced hurdles in expressing needs during and beyond disasters/EWEs.

Participants with hearing disabilities living in rural areas (Thanh Chuong and My Duc districts) do not know sign language. They only know body language and rely on their family members to interpret for them. For Lan (PA08.2), her challenge stemmed from shyness and fear of communicating with strangers, while her family does not always trust her or is willing to understand what she needs. For Van, a 41‐year‐old female with speech and hearing disabilities from Nghe An province (PA08.3), she must rely on her husband to communicate her needs since she does not know sign language.

People with cognitive disabilities or those having mental health impairments all confronted this challenge. The father of a 32‐year‐old male with intellectual and multiple disabilities (PA23) observed: ‘Whether they can hear or not, I don't know. They don't know how to even express hunger. They just don't call for dad or mum’.

#### 3.2.4 Evacuation and mobility hurdles

Navigating hazards to evacuate was found to be mostly relevant to people with mobility and visual disabilities. Current research highlights the difficulties faced by individuals with mobility disabilities during evacuation from a home or workplace, notably, limited mobility, unsafe conditions in rooms caused by insecurely installed furniture and equipment, and vulnerability due to glass partitions and hanging ceilings (Alexander, Gaillard, and Wisner, 2012; Jodoin, Ananthamoorthy, and Lofts, 2020). Also noted are challenges with setting up tents, managing limited space, and controlling the temperature inside tents due to exposure to the environment (Aryankhesal, Pakjouei, and Kamali, 2018).

Our fieldwork found that people with mobility, visual, and cognitive disabilities faced major hurdles during evacuation—the exception was people with hearing disabilities. For instance, the challenge for people with mobility and visual disabilities is not just derived from their inability to move quickly in emergencies, but also from their family's lack of means of escape, such as a boat. A 35‐year‐old woman with mobility disabilities in My Duc district (PH13) captured this when she said: ‘Without a boat or basket, we couldn't really go outside. Most of us moms and kids stayed home waiting for the water to recede’. The same situation applies to visually disabled persons living in urban low‐lying areas in Vinh city, Nghe An province. A 51‐year‐old woman with visual disabilities living along the La River (PA09) recounted the severity of flooding: ‘When it flooded, some areas were so bad you needed a boat. It was because the pumping station at Bến Thuỷ broke down, flooding everywhere around here’.

People with intellectual disabilities are highly dependent on their caregivers, who are often their parents or family members. For those who also have mobility impairments, moving and caring for them during evacuation are even more challenging tasks. A prime example is a 32‐year‐old male from Nghe An who has had a mobility disability since birth and, at the age of five, developed encephalitis after a sudden illness. His father (PA23) told us:

*The flood made it difficult to move him to the temporary site at the primary school. We [my wife and I] had to stay at home because the shelter was not spacious enough. Since my whole family couldn't stay at the shelter together, we felt the family was divided*.

#### 3.2.5 Decreased sense of belonging and isolation

We found that disasters/EWEs have a great impact on people with disabilities' social relationships (except for people with cognitive disabilities). At such times, there is a reduction in or a loss of communal activities with family or neighbours, leading to a decreased sense of belonging and support for people with disabilities (Bourke et al., 2017). A 40‐year‐old woman with mobility disabilities (PA07) reported that floods often create movement restrictions that have limited her participation ‘in training or community activities’. And a 44‐year‐old woman with mobility disabilities observed that floods and storms only affected her outside relationships: ‘It just limits visits and socialising with friends. My relationship with my family isn't affected’.

The literature shows the role of climate‐related disasters/EWEs in generating prejudice and violence towards people with visual disabilities (Gomes, Marchezini, and Sato, 2022); however, we did not find evidence of this in our qualitative interviews. Only one participant said that they had experienced some shouting by their husband during isolation. Yet, we confirm that most people with visual disabilities have found themselves isolated during floods or storms since they prefer to stay at home at these times. A 40‐year‐old female with mobility disabilities from My Duc district, Hanoi (PH16), stated: ‘When storms and floods came, I only stayed at home, I didn't want to go anywhere’.

The literature review indicated social isolation of hearing‐disabled people; however, our fieldwork did not confirm this. Rather, our research found no evidence of deaf people facing social prejudice or social isolation. A 21‐year‐old female with hearing disabilities living in Cau Giay district, Hanoi (PH21.1), confirmed this point: ‘Most of us deaf individuals do not feel abandoned since we're cared for by our families, with young people being able to maintain online connections with friends’. Yet, she observed that relationships with other deaf friends can change due to emergencies, pointing out that: ‘Heat makes my [deaf] friends irritable, quick‐tempered, and sometimes disrespectful towards me’.

#### 3.2.6 Increased risk of getting sick

The impact of disasters/EWEs on physical health was not well‐documented by types of disabilities. In our review, the negative effects on physical health were mostly associated with people with mobility disabilities. For instance, health risks in emergencies often stem from inability to access the usual health‐assistive devices, such as talking equipment and wheelchairs, or a loss of basic amenities, such as power, creating life‐threatening situations for those with an impaired thermoregulation (Quaill, Barker, and West, 2019). While the challenge of losing equipment was not prominent in our interviews, since not every participant had a wheelchair, we did observe that a lack of access to key facilities in temporary shelters often had a negative bearing on people with mobility disabilities. For instance, a 65‐year‐old man with mobility disabilities in Nghe An province (PA02) recalled:

*Because of the flooding, my family had to move me to a friend's house to stay for a week waiting for the water to go down. That time, there was no clean water or electricity*.

Women with disabilities are even more vulnerable due to their unique need for hygienic conditions, which are often absent or limited in a time of evacuation. A 56‐year‐old woman with a mobility disability from Vinh city, Nghe An province (PA01), reflected on this matter: ‘Aches, difficulty sleeping, and issues with personal hygiene and mobility’.

Weather changes and EWEs often reveal the extent to which people with mobility disabilities are vulnerable. In the words of a 34‐year‐old man with a mobility disability from My Duc (PH19):

*Generally, with the weather changing like this, I've been feeling off, getting sick easily. We're weak, often have headaches, frequently sick. After the flood water goes down, there are diseases, conjunctivitis, a lot of hygiene‐related infections. Weather changes affect my respiratory system*.

Few studies have documented the impact of disasters/EWEs on the physical health of people with visual and cognitive disabilities. While no information was found in our review of the impact of EWEs on people with visual disabilities, our fieldwork found that an extreme change in weather often exacerbates this group's health problems. For instance, a 31‐year‐old woman with visual disabilities from Hanoi (PH05) shared her experience: ‘I get sick easily, especially when it's about to rain. I feel dizzy and just unwell’. Meanwhile, only one of the studies reviewed, by Jodoin, Ananthamoorthy, and Lofts (2020), reported on people with a mental health impairment in EWEs: they are three times more likely to die in a heatwave. Our fieldwork paints a bigger picture: people with cognitive and intellectual disabilities are extremely susceptible to weather changes, let alone disasters/EWEs. A prime example is a 52‐year‐old male with multiple disabilities (including cognitive and mobility impairments) in Vinh city, Nghe An province (PA05), who found it difficult to bear a heatwave that occurred from June–September 2023. In this case, temperatures often exceeded 38–40°C from 11:00 to 13:00, making it unbearable to be outside. When he attempted to go out, he was prone to heatstroke and headaches. Further complicating matters is his responsibility to care for his two sons who also have intellectual disabilities, in the absence of his wife who was recently rendered paralysed by an accident.

#### 3.2.7 Increased risk of developing mental health and behavioural disorders

The impact on mental health was mostly associated with people with cognitive or intellectual disabilities. The rapid evidence review highlighted that the mental health of this group is highly sensitive to light, sound, and surroundings during disasters (Ronoh, Gaillard, and Marlowe, 2015); people feel uncomfortable in crowded places (Mendis et al., 2023), and often have increasing anxiety during disasters (Stough, 2009; King et al., 2019). Our fieldwork revealed stories of family members who faced challenges in supporting their children with intellectual disabilities in adversity. The wife of a 52‐year‐old man with intellectual and multiple disabilities living in Thanh Chuong district, Nghe An province (PA21), recalled that during heavy rains a few years ago, her children were taken to the evacuation site, but she had to stay at home with her husband. She said:

*It's difficult for him to move around so we stayed at home. Whenever he had an episode, he started breaking doors, then we had to lock his wrists*.

In extreme cases, the risk of mental disturbance increases in families with multiple members with intellectual disabilities. Hot weather, for instance, is reported to heighten irritability and can cause potential injuries. A 52‐year‐old male with multiple disabilities in Vinh city (PA05) remarked on the difficulty of caring for his two intellectually disabled sons in a heatwave:

*One ate, the other destroyed things, going crazy. Like when I came home between 11–12 o'clock, it's very hot, and their destruction happened*.

These stories point up the emotional susceptibility of individuals with intellectual disabilities in a time of crisis.

Our fieldwork also reveals the impact of disasters/EWEs on the mental health of people with visual disabilities, although no evidence was found in the literature reviewed. A 42‐year‐old man with visual disabilities in Vinh (PA10) underscored:

*Having a disability makes me feel so weighed down with self‐doubt. Extreme weather was tough enough before, but now, it feels like I'm just a dead weight*.

#### 3.2.8 Disrupted livelihood and loss of income

The impact of disasters/EWEs on economic opportunities and income is evident among people with mobility disabilities, often persisting into the post‐disaster period (Van Willigen et al., 2002; Bourke et al., 2017). Our qualitative interviews produced a great amount of information indicating this type of impact, not just on people with mobility disabilities, but also on people with visual and hearing disabilities. No people with cognitive disabilities reported being employed at the time of the interviews.

The livelihoods of the people with hearing disabilities interviewed included sign language interpreters/teachers, grocery sellers, tailors, and freelance construction workers, while people with visual disabilities often worked as farmers or masseurs. Weather extremes, including flooding and prolonged hot days, can significantly affect their businesses. A 38‐year‐old male masseur with a visual disability in Hanoi (PH10) stated:

*Storms hit my business hard. Who's going to come out in that weather? My job's heavily dependent on good weather*.

Yet, disasters/EWEs did not just affect livelihoods, but also living standards. During disaster events that contaminate drilled water, the need to filter and use rainwater becomes more urgent. A 28‐year‐old female with hearing and speech disabilities from Thanh Chuong district, Nghe An province (PA20), put the cost at ‘about VND 5.5 million’ per water tank.

People with mobility disabilities worked in a more diverse set of livelihoods, including barbers, houseworkers, bakers, mechanics, and livestock farmers. One 34‐year‐old male barber with mobility disabilities in Cau Giay district, Hanoi (PH19), recalled:

*The flood ruined a lot like furniture, and stuff for my barbering had to be moved first. Otherwise, I had to throw everything else out. When it rains heavily or there's a power outage during hot weather, my barber and shampoo shop often have to close. My income dropped to just a couple of million [VND] a month*.

## 4 DISCUSSION

Our study makes some significant contributions to the topic of people with disabilities' vulnerabilities in disasters/EWEs. We provide a more holistic view of disaster impacts across various disability types (mobility, visual, hearing, and cognitive/intellectual impairments). This is significant as many previous studies have focused primarily on one type of disability, often mobility (Stough, 2009; Gaskin et al., 2017). By combining a rapid evidence review and qualitative data, we found that people with mobility disabilities faced the compounding effects of inadequate infrastructure (such as the lack of a boat for evacuation) and increased risk of getting sick. People with visual disabilities experienced negative mental health impacts and income loss in certain professions (including massage therapy in urban areas). People with hearing disabilities had a strong reliance on family members for disaster information, rather than official channels, but reported that social isolation might not be as prevalent as suggested in the literature (Cooper et al., 2021), with many maintaining connections through online platforms. People with cognitive disabilities depended entirely on family support, especially in managing their behavioural and mood changes during EWEs, and their mental health was more susceptible to changes in weather. Furthermore, the understudied group, people with multiple disabilities (combining mobility, hearing, and intellectual impairments, for instance) were even more susceptible to changes in weather owing to their complicated health conditions and, sometimes, childcare responsibilities. Differences in vulnerabilities to disasters/EWEs among people with disabilities imply the need to consider all types of (dis)abilities and functioning when designing social, economic, political, health, and built environments (Kelman and Stough, 2015).

Several key impacts that require further research are not discussed much in the existing literature. Expanding on the findings of Aldrich and Benson (2008), we identified significant disruption to the livelihoods of people with mobility, visual, and hearing disabilities who work as masseurs, tailors, and barbers. Additionally, we have provided more insights into the health impacts of disasters/EWEs on people with mobility, visual, and especially intellectual disabilities, ranging from physical injuries to mental health issues, such as stress, depression, and the exacerbation of existing mental and behavioural disorders and aggression (Stough, 2009; Gaskin et al., 2017). Consequently, it is crucial that disaster management strategies incorporate mental health support tailored to people with different types of disabilities.

Experiencing significant disaster impacts implies different systemic layers of the root causes of disaster vulnerability among people with disabilities (Nguyen‐Trung, Matthewman, and Uekusa, 2023). For example, our theme concerning the lack of disabled‐friendly early warning systems and accessible communication products and languages, especially for people with hearing and visual impairments, observed in the two areas under review, echoes the insights in the current literature (Alexander, Gaillard, and Wisner, 2012; Stough and Kelman, 2018; Cooper et al., 2021; Habibisaravi et al., 2022). Like Battle (2015), we also observed that people with intellectual and multiple disabilities faced greater challenges not just in receiving weather forecasts, but also in understanding early warnings and communicating their needs during adversity. We agree, though, that those challenges cannot be seen as the outcome of just the limited individual capacity of people with disabilities. Rather, they are also due to the lack of suitable cultural, social, economic, and political support factors, highlighting the significance of the capability approach in disability research (Ton et al., 2020). In other words, people with disabilities' vulnerability to disasters/EWEs, like that of other groups, should be seen as a socially‐constructed outcome—disasters can be avoided if society helps them to reduce this vulnerability and build resilience (Kelman, 2020; Forbes‐Mewett and Nguyen‐Trung, 2019). For instance, the lack of accessible risk communication products in sign language can be attributed to the fact that ‘Việt Nam does not have formal signed‐spoken language interpreter training programs, nor systems for assessment and credentialing, so all services are delivered through largely self‐ and community‐taught intermediaries’ (Cooper et al., 2021, p. 8). This is especially true for people with hearing disabilities: in Nghe An province, this group lacks literacy in sign language and has few opportunities to learn this language at the local level owing to the absence of classes and teachers; such opportunities are often accessible in Hanoi. Similarly, people with cognitive disabilities' family members and caregivers have limited experience in providing timely risk warnings and proper care in a time of disaster, since disability inclusion is not part of disaster management training.

In sum, we need to employ a critical disaster studies perspective to scratch beneath the surface of disaster impacts to reveal the root causes of unsafe conditions embedded in the social, economic, political, and cultural systems of the affected society (Wisner et al., 2004; Oliver‐Smith, 2022). By so doing, we champion the need for tailored, inclusive planning strategies in DRR and climate change adaptation that address the specific needs of various disability types in numerous settings, as suggested by Bourke et al. (2017) and Aryankhesal, Pakjouei, and Kamali (2018). Without such an intervention, people with disabilities, like other groups vulnerable to disaster, could be locked in a permanent state of vulnerability (Nguyen‐Trung, Uekusa, and Matthewman, 2024).

## 5 CONCLUSION

The findings of our study underscore the significant vulnerability and unique challenges that people with various types of disabilities in Vietnam face in the context of disasters/EWEs. We identified the following related impacts arising from their pre‐existing vulnerabilities: barriers to accessing disaster risk information and warnings; difficulties in understanding emergencies; challenges in communicating needs; evacuation and mobility hurdles; decreased sense of belonging and isolation; increased risk of getting sick; increased risk of developing mental health and behavioural disorders; and disrupted livelihood and loss of income. Preparedness for climate change and disasters/EWEs among people with disabilities is alarmingly inadequate, marked by a lack of inclusion in planning and adaptation. This is evidenced by inadequate access to disability‐friendly early warning systems and risk communication products and channels. The Government of Vietnam in particular, but also those of other disaster‐prone countries, should engage OPDs' experts in updating disaster preparedness and response plans and tailor early warning messages concerning disasters to the needs of different types of disabilities.

## DISCLAIMER

The authors have no relevant financial or non‐financial interests to disclose. This work reproduces, with permission, materials from our research report funded by the AWP.

## ETHICS STATEMENT

This paper reports analysis of primary data. The ethics of data collection and analysis were approved by the Monash University Human Research Ethics Committee.

## FUNDING

The authors acknowledge the Australian Water Partnership (AWP) for providing funds to conduct the research that led to this article. The AWP is supported by the Australian Government and managed by the eWater Group.

## SUPPORTING INFORMATION

Additional supporting information may be found online in the Supporting Information section at the end of the article.

## Supporting information


Data S1

## Data Availability

The data that support the findings of this study are available on request from the corresponding author. The data are not publicly available due to privacy or ethical restrictions.

## Endnote

^1^
All supplementary materials can be found online in the Supporting Information section at the end of the article.
