# Supplementary material for: Vulnerabilities of people with different types of disabilities in disasters: a rapid evidence review and qualitative research
Source: Disasters. 2025 May 12;49(3):e12686. doi: 10.1111/disa.12686 (PMC12067366; doi:10.1111/disa.12686)
Supplement: Supplementary file 1 — Data S1 [file DISA-49-e12686-s001.docx]

## Appendix 1: Review Protocol

1. Objective

The objective of this rapid review is to assess the impact of climate-related events on people with disabilities, focusing on the variability of impacts across different types of disabilities in developing countries, particularly Vietnam.

2. Review Question

*How does the impact of climate-related disasters and extreme weather events (EWEs) vary among individuals with different types of disabilities in the context of Vietnam?*

3. Eligibility Criteria

Inclusion Criteria:

Type of publication: Primary studies, reviews, reports.

Language of publication: English only.

Date range: 2017 – 16/10/2023.

Setting: All countries.

Study focus: Studies that examine or discuss the impact of climate-related events on people with disabilities.

Exclusion Criteria:

Studies not focused on the relationship between climate-related events and disabilities.

Non-English studies.

Grey literature not falling within the inclusion criteria (e.g., blogs, opinion pieces).

4. Information Sources

The following databases were used to identify relevant literature:

Scopus: Academic database.

Google Scholar: A modified search string was used to screen the first 105 citations, screened by one reviewer.

5. Search Strategy

A search string was developed by a library information specialist (VD) in collaboration with the research team, based on background documents. The search string used in the Scopus database and a modified version for Google Scholar can be found in Appendix 2.

6. Screening Process

Title and Abstract Screening: Performed by one researcher (CN) independently. The researcher kept a log of decision-making to support transparency and consistency in the process.

Full-Text Screening: Independently carried out by the same researcher. Records of the decision process were maintained to ensure the reliability of the screening process.

Software: No specific software was used for the screening, but results were managed using Excel.

7. Data Extraction Process

Data Extractor: One researcher was responsible for data extraction.

Data Extraction Items: Information extracted included:

Study design.

Sample characteristics (e.g., types of disabilities, age, gender).

Geographic location of study.

Key findings related to climate-related vulnerabilities.

Recommendations for climate adaptation and DRR related to PwDs.

8. Quality Appraisal

Due to time and resource constraints, quality appraisal of individual studies was not conducted. This limitation should be considered when interpreting the findings of this review. The review findings may be biased due to the omission of formal quality checks.

9. Data Synthesis

The data from the included studies were synthesized thematically, focusing on common trends in climate vulnerability across different disability groups, types of climate-related events, and geographic locations.

10. Limitations

Database Restriction: The review was restricted to two databases (Scopus and Google Scholar), which may limit the comprehensiveness of the search.

Language and Time Constraints: The review only included studies published in English and limited the search to the past five years.

Quality Appraisal Omission: The lack of a formal quality appraisal process introduces potential biases.

11. Bias Management

Biases from the lack of quality appraisal were mitigated by cross-referencing review findings with data from the fieldwork. This triangulation process helped verify findings and reduced the impact of possible biases introduced by the limited scope of the review.

12. PRISMA Flow Diagram

The review process follows the PRISMA framework for systematic reviews, detailing the number of records identified, screened, and included in the final synthesis. The PRISMA diagram can be found in Appendix 3, adapted from Page MJ et al. (2021).

## Appendix 2: Search Strings

### Scopus database

Search strings:

TITLE-ABS-KEY ( "climate change" OR disaster* OR "climate governance" OR "water

insecurit*" OR "water securit*" ) AND TITLE-ABS-KEY ( disabili* OR disable* OR impair* OR incapacitat* OR "physically challeng*" OR "physically disadvantage*" OR "mentally disadvantage*" OR vulnerab* OR marginali* OR "adaptive capacit*" ) AND TITLE-ABS-KEY ( people OR person* OR individual* OR communit* OR population* ) AND TITLE-ABS-KEY ( inclusi* ) AND PUBYEAR > 2016 AND ( LIMIT-TO ( DOCTYPE , "ar" ) OR LIMIT-TO (

DOCTYPE , "re" ) OR LIMIT-TO ( DOCTYPE , "ch" ) OR LIMIT-TO ( DOCTYPE , "cp" )) AND ( LIMIT-TO ( LANGUAGE , "English" ) )

Results (16/10/2023): 787 records

### Google Scholar

Search strings:

"climate change"|disaster*|"weather"|"water insecu*"|"water security" AND disabili* AND

impair*|natural

Result (16/10/2023): first 105 records

## Appendix 3: PRISMA Diagram & Summary of Included Studies in the Review


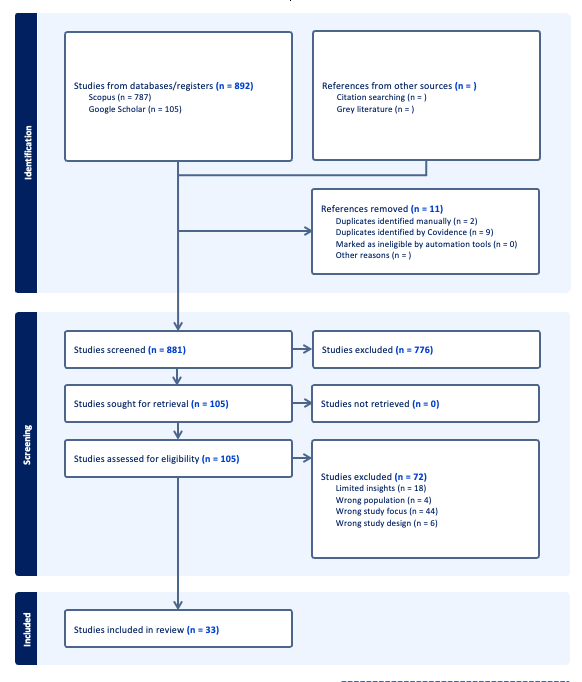


A total of 33 studies were included in the data extraction and synthesis phase. Key characteristics include:

- Types of disabilities: Physical (N = 3), visual (N = 2), deaf (N = 2), communication (N = 1), combination of disabilities (N = 25).
- Research designs: Systematic reviews (N = 5), narrative reviews (N = 16), qualitative studies (N = 12).
- Geographic distribution: Studies conducted in Australia (1), Cambodia (1), Brazil (1), Iran (1), New Zealand (2), South Korea (2), Solomon Islands (1), Uganda (1), USA (1), and Vietnam (1).
- Age focus: Eight studies focus on children with disabilities, while four target older adults.
- Gender-specific impact: Three studies discuss the impact of climate change specifically on women with disabilities.
- Disability focus: Mobility impairments were the most discussed disability (N = 23), followed by hearing and cognitive impairments (N = 19), vision impairments (N = 15), communication impairments (N = 8), and self-care impairments (N = 3).

## Appendix 4: Purposeful stratified sampling strategy

The purposefully stratified sampling strategy helps design our sampling strategy to these two locations. The following steps outlined our approach in executing this strategy:

- Choosing two provinces whose social contexts and climate change vulnerabilities are assumed to be different.
- In each province, select one urban district and one rural district. In Hanoi, we chose Cau Giay and My Duc districts. In Nghe An province, we chose Vinh city and Thanh Chuong district.
- In each district, we purposefully chose PwDs for our semi-structured interviews so that the sample achieves a relative balance in terms of disability types, gender, age groups.
  - Regarding disability types, we focused on vision, hearing, intellectual, mobility types. We ended up having participants with multiple disabilities. We aligned our work with the 2016 Vietnam National Survey on People with Disability (GSO, 2016), which used the Washington Group Extended Set (WG-ES) questionnaire to guide the identification of PwDs. This WG-ES is designed to identify different types of disabilities: vision, hearing, mobility, cognition, communication, selfcare, psycho-social, and multiple types. In this study due to the limited capacity in recruitment and time constraints, we only focused on four main types of disability: visual, hearing, mobile, and intellectual. In addition to the use of WG-ES in our semi-structured interviews with PwDs, we also incorporated one self-declaring question that allowed PwDs self-declare their types of disability.
  - Regarding age groups, we aimed to collect information from a diverse age groups, ranging from 18 to 60 and above.
  - Regarding gender, we designed our interview question to involve people with the sex other than male or female, although eventually we only had records of the two latter sexes.
- In addition, participants chosen must meet the following criteria:
  - Having lived or worked in the selected location (district) in the past 5 years or so.
  - Must be at least 18 years old or above
  - Having capability to understand and respond to our interview questions. Or if they do not have this capacity, they must have their family members or carers to support them join and respond to our interviews.
  - Having experienced some forms of weather extremes or natural hazards.
- DP Hanoi’s researchers worked with their local partners whose are Associations of People with Disabilities in Cau Giay, My Duc districts, Nghe An province, Vinh city and Thanh Chuong district to conduct recruitment of PwDs. These local partners supported in recruiting and approaching PwDs who meet the sampling criteria.
- During the fieldwork, in addition the recruitment by DP Hanoi and local stakeholders (i.e., organisation of disabled people in Nghe An province), the research team also flexibly used snowballing technique in recruiting PwDs to ensure the diversity of our sample. We asked our research participants to introduce to their colleagues or friends who meet the criteria and invite them to join our interviews.

## Appendix 5: Topics and Structures of Interview Guide with PwDs

The topics and structures of the Interview Guide are presented below. The full Interview Guide will be provided on request and subject to the approval by DP Hanoi, Water Sensitive Cities Australia (WSCA), Monash University, and the Australian Water Partnership (AWP).

The interview guide consists of two parts:

The first part includes key indicators including:

- Key socio-demographic characteristics: gender, age, place of residence, marital status, living with, education, literacy, occupation, workplace, income, poor household, association, training, mobility.
- Disability Type and Allowance: cause, disability certificate, disability support, level of disability, satisfaction, level of difficulty according to type of disability.
- These indicators are not designed to conduct complex modeling. Instead, they supported the identification of PwDs, building their profiles, and case-based analysis of qualitative data.

The second part of the Interview Guide focuses on the experience of PwDs during the events of extreme weather.

Vulnerability and interaction with climate change:

- Access to Water: water source, fetching water, water sufficiency for daily use, during disasters/EWEs, clean water, why clean/not clean, concerns, water treatment.
- Disasters/EWEsWeather Changes: natural disaster, most memorable natural disaster, year.
- Access to Information: daily information channels, information form, informer, level of proactivity in accessing early warnings, receiving information, difficulty in accessing information according to type of disability.
- Infrastructure Impact and Evacuation: infrastructure, healthcare, evacuation shelter, difficulty in evacuation, infrastructure facilities.
- Access to Water during Disaster: access to water during disaster, consequences.
- Health including Mental Health: assistive devices, health, including mental health.
- Support and Family, Community Relations during Disaster: support, access to support, special support for PwDs, family relationships, disability association, other social supports, community relations, support during evacuation, discrimination, gender violence.
- Post-Disaster Support: recovery after disaster, livelihood and income.
